# Chemical Inductor

**DOI:** 10.1021/jacs.2c00777

**Published:** 2022-03-22

**Authors:** Juan Bisquert, Antonio Guerrero

**Affiliations:** † Institute of Advanced Materials (INAM), Universitat Jaume I, Castelló 12006, Spain; ‡ Yonsei Frontier Lab, Yonsei University, Seoul 03722, South Korea

## Abstract

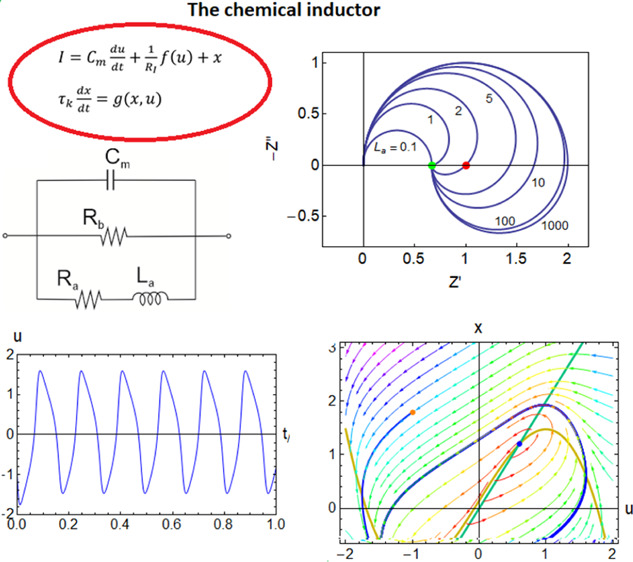

A multitude of chemical, biological, and material systems present an inductive behavior that is not electromagnetic in origin. Here, it is termed a chemical inductor. We show that the structure of the chemical inductor consists of a two-dimensional system that couples a fast conduction mode and a slowing down element. Therefore, it is generally defined in dynamical terms rather than by a specific physicochemical mechanism. The chemical inductor produces many familiar features in electrochemical reactions, including catalytic, electrodeposition, and corrosion reactions in batteries and fuel cells, and in solid-state semiconductor devices such as solar cells, organic light-emitting diodes, and memristors. It generates the widespread phenomenon of negative capacitance, it causes negative spikes in voltage transient measurements, and it creates inverted hysteresis effects in current–voltage curves and cyclic voltammetry. Furthermore, it determines stability, bifurcations, and chaotic properties associated to self-sustained oscillations in biological neurons and electrochemical systems. As these properties emerge in different types of measurement techniques such as impedance spectroscopy and time-transient decays, the chemical inductor becomes a useful framework for the interpretation of the electrical, optoelectronic, and electrochemical responses in a wide variety of systems. In the paper, we describe the general dynamical structure of the chemical inductor and we comment on a broad range of examples from different research areas.

## 1 Introduction

The familiar inductor element used in the analysis of electromagnetic circuits was discovered by Faraday. A variable current *I*(*t*) that passes through an inductance *L* generates a voltage *u*_L_ opposing the increase in current
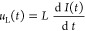
1

When a voltage source *V*_0_ applied to an inductor with a series resistance *R*_s_ is switched on, the current risen to the final value *V*_0_/*R*_s_ is delayed by a characteristic time *L*/*R*_s_. By taking the Laplace transform of 1 in terms of the variable *s* = iω, where ω is the angular frequency, the impedance of the inductor takes the form
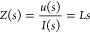
2

**Figure 1:**
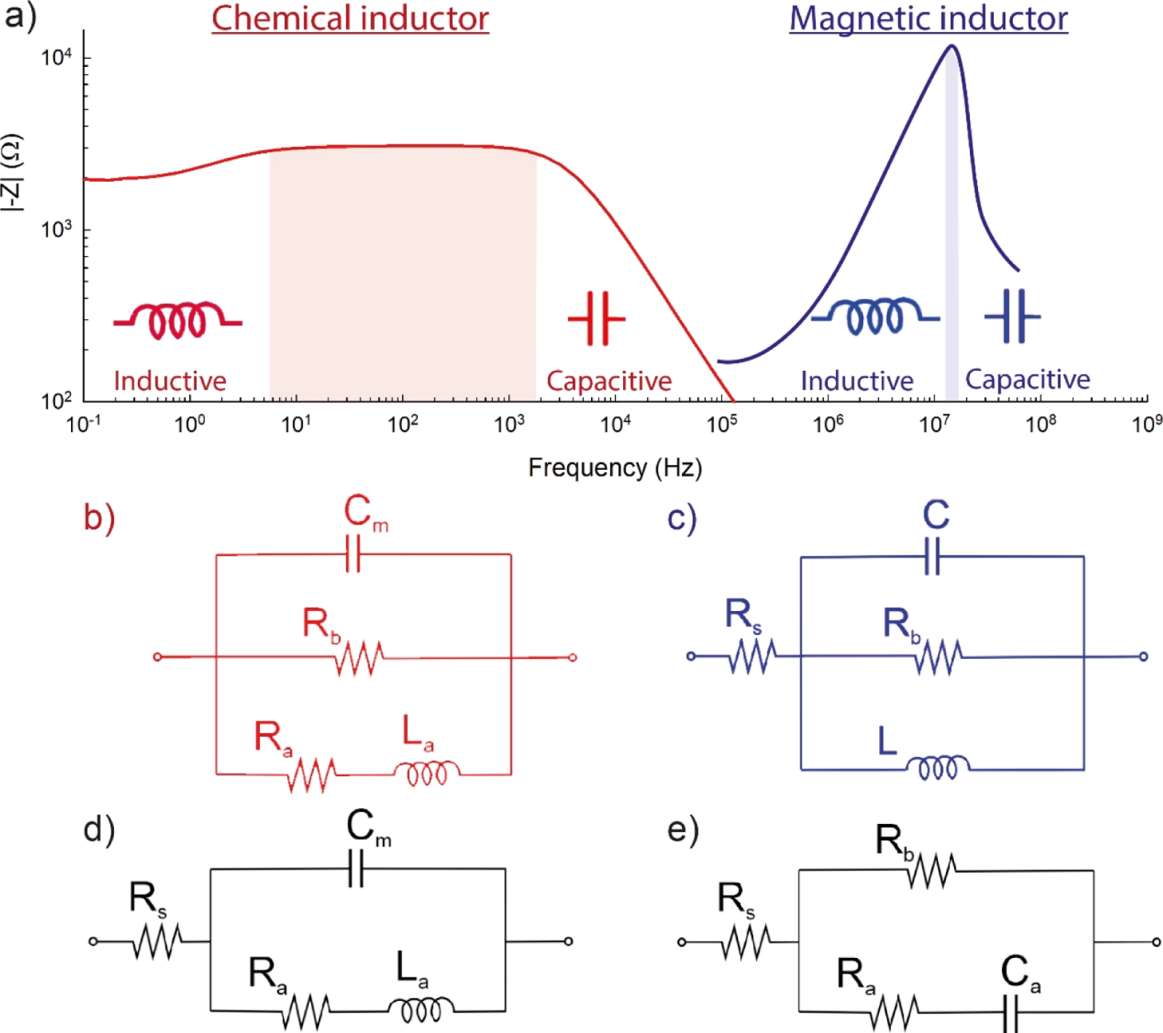
(a) Comparison of the impedance response of chemical and magnetic inductors. Red and blue lines correspond to the circuits of the same color below. Equivalent circuits for (b) chemical inductor under voltage control (parallel mode), (c) electromagnetic inductor, (d) chemical inductor circuit under voltage control (series mode), and (e) current–control model, including a negative capacitance.

These features are usually associated with the phenomenon of electromagnetic induction, in which a variable magnetic flux intersects a coiled wire. An inductive response is obtained in the impedance of magnetic materials, as shown on the right side of Figure 1a (blue section). However, there is a totally different class of phenomena that produces the same temporal dynamics and the same impedance as the typical coil inductor, as shown in eqs 1 and 2, but does not arise from an electromagnetic origin. It is indicated by the red section on the left side of Figure 1a.

Chemical and electrochemical systems,^1^ neurons,^2,3^ and optoelectronic semiconductor devices like solar cells^4^ and organic light-emitting diodes (LEDs)^5^ contain an inductive behavior in the small-signal ac impedance (an arc in the fourth quadrant of the complex plane), a negative spike component in the time-transient decays, and, in some cases, an oscillatory response to stimuli. These features form a general dynamic behavior across different types of systems rather than a specific physical mechanism. It is a futile exercise to search for a microscopic coil in these systems. In this paper, we describe the generic behavior that we call a “chemical inductor”, which arises from two coupled processes. The first responds rapidly to an external stimulus, and the second is slow and delayed with respect to the first one.

Historically, the observation of inductive processes is well recognized in the dominant models of neuroscience, such as the Hodgkin–Huxley (HH) paradigm.^3^ In this model, and in hundreds of similar derived mechanisms,^6,7^ the ionic conductivity across the ionic channel in the membrane is delayed by a voltage- and time-dependent conductivity function. Emerging solar cells and lead halide perovskite electronic devices represent examples in which a strong inductive response is measured in impedance spectroscopy analysis.^4,8,9^ The electronic transport under an applied voltage is a fast process, and interactions of mobile ions with contacts is a slow process that delays the overall electrical current. The main difference between the properties of the chemical and magnetic inductor can be observed in Figure 1. A magnetic inductor such as a ferrite inductor is a fast response system in which the rise of the impedance at an increasing frequency of eq 2 is suppressed by a parasitic capacitance, as shown in Figure 1c, causing resonant frequencies of 10–100 MHz. The inductor element stands alone in the equivalent circuit. The chemical inductor is typically much slower with a peak response in 1–10 Hz, and the inductor branch contains a resistance *R*_a_ in series with the inductor *L*_a_, as shown in the equivalent circuit of Figure 1b that is central in this work. This chemical inductor structure is familiar in many research fields that use impedance spectroscopy and time-transient response, but a general description has not been given and separate mechanisms are searched each time.

In this paper, we aim to clarify the structure of this feature in terms of a minimal dynamical model that can be found in very different systems, with variables that carry distinct physico-chemical interpretations. We use the methods of equivalent circuits to represent impedance spectroscopy data and obtain an interpretation of the system.^8,10,11^ An important feature of the practical analysis of impedance spectroscopy is that the circuit elements change exponentially with the voltage. Hence a variety of spectra are possible in a single system, according to the evolution of the individual elements. Therefore, the equivalent circuit that is valid over a wide voltage range is an important tool to comprehend the mechanisms that form a system’s response.

**Figure 2:**
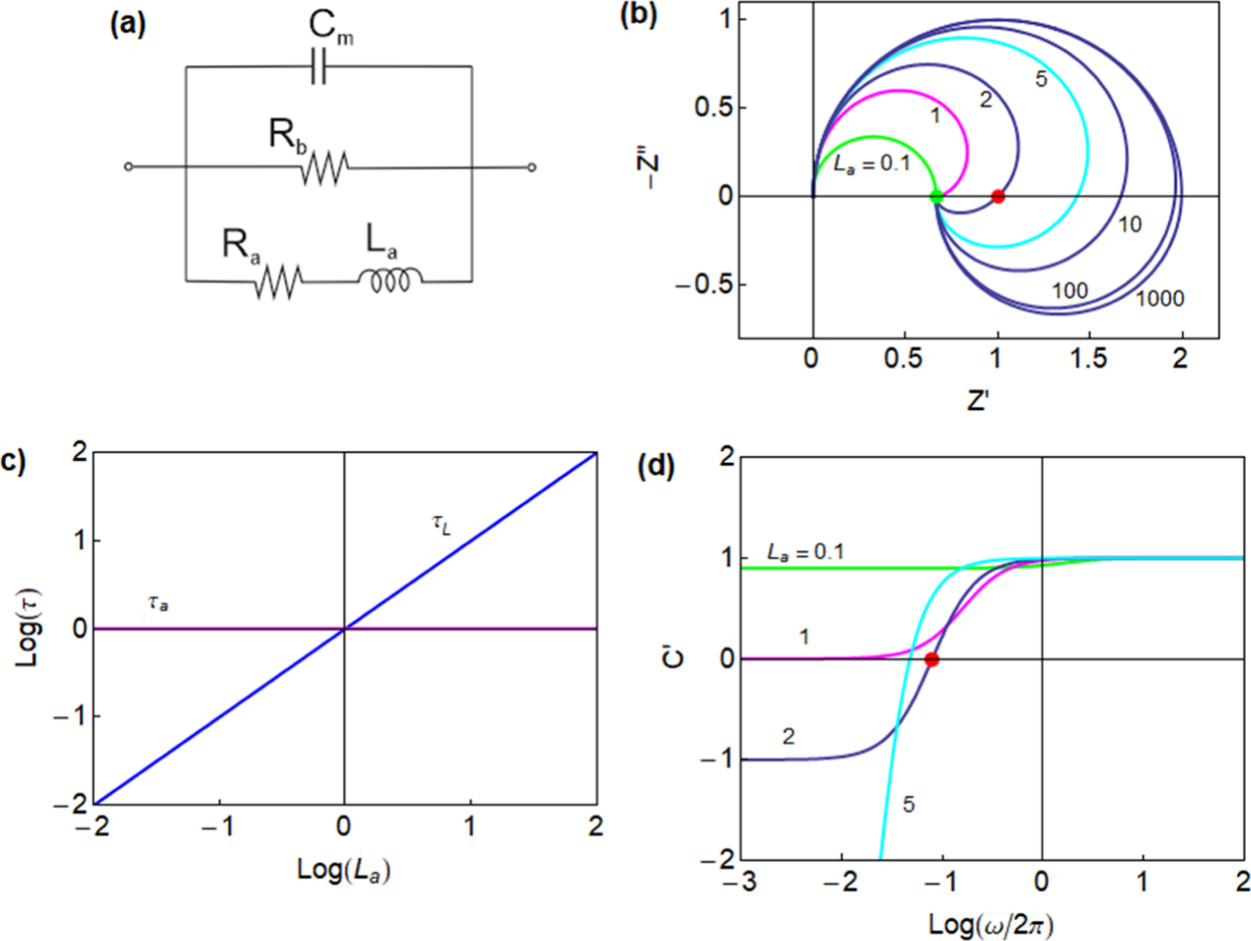
(a) Equivalent circuit corresponding to eq 8. (b) Set of impedance spectra generated for *C*_m_ = 1, *R*_a_ = 1, *R*_b_ = 2 and *L*_a_ as indicated. The green dot is the dc resistance at ω = 0, and the red dot is the resistance at the intercept when the spectrum crosses the real axis *Z*′ at ω = ω_c_. (c) Time constants τ_a_ = *R*_a_*C*_m_ and τ_L_ = *L*_a_/*R*_a_ for a varying inductance. (d) Representation of the real part of the capacitance as a function of frequency. The red point indicates the crossing of the horizontal axis.

**Figure 3:**
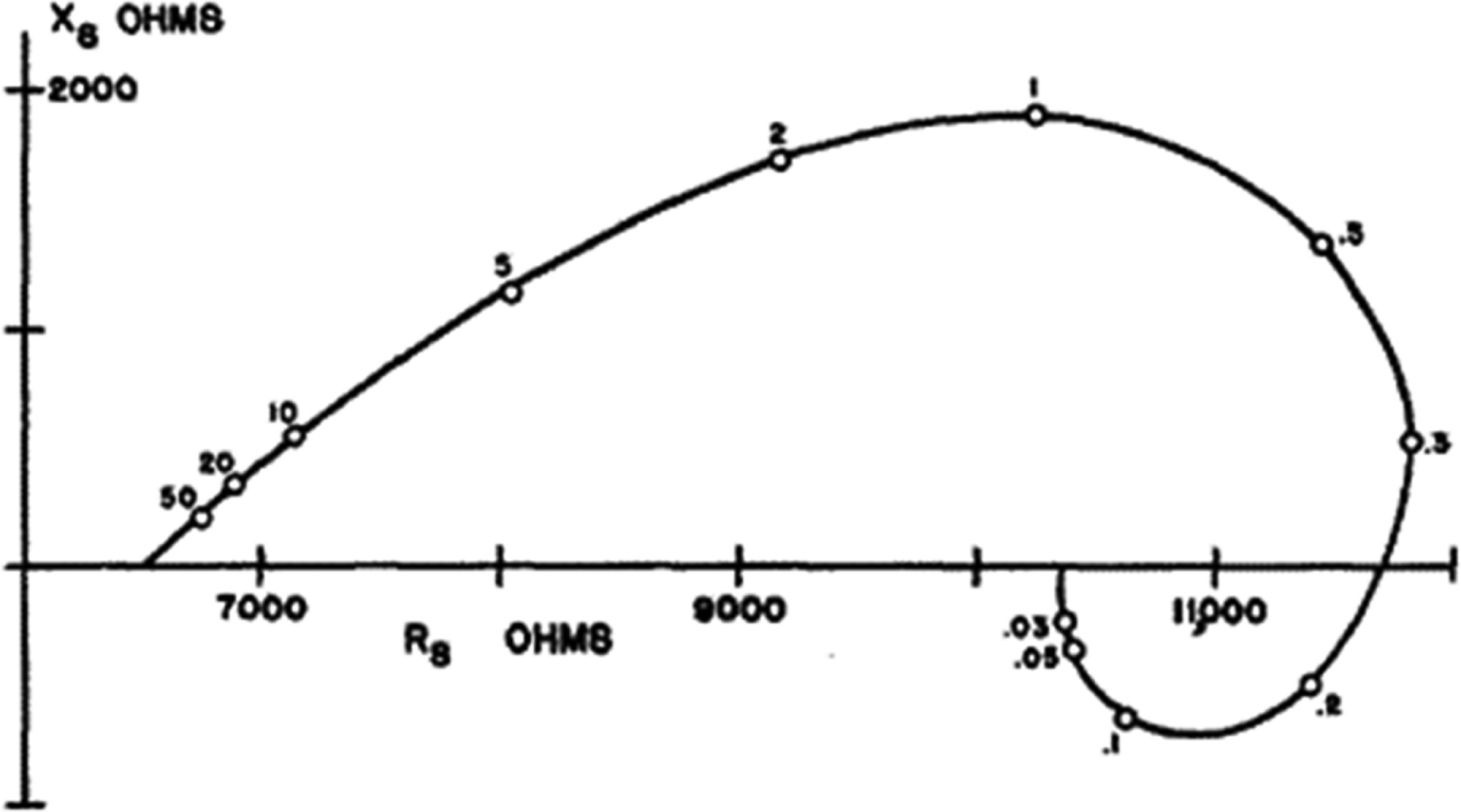
Complex plane plot of the impedance of the squid giant axon. The frequencies in kHz are indicated. Republished with permission of the Rockefeller University Press, from Cole, K. S., and Baker, R. F. *Journal of General Physiology***1941**, *24*, 771–788. Copyright Rockefeller University Press (1941).

**Figure 4:**
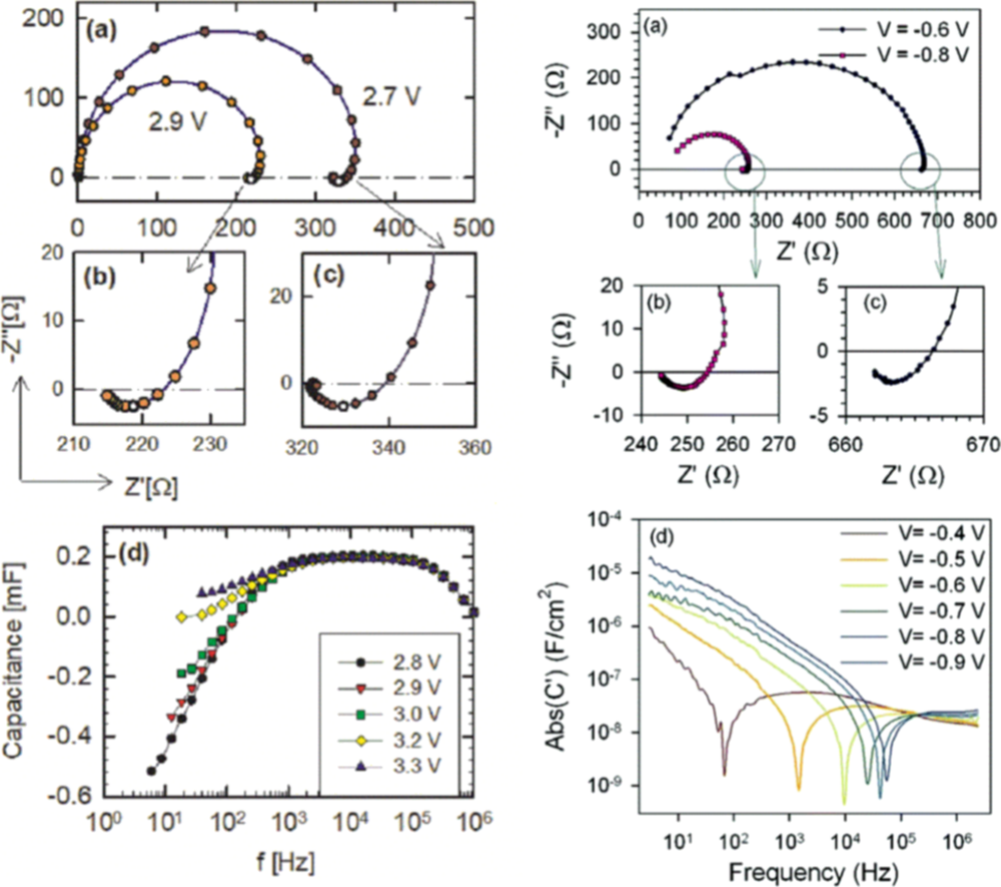
Left column. Results of the measurement of an ITO/PEDOT/superyellow/Ba/Al organic LED device. (a) Impedance plots for different bias voltages. (b,c) shows a magnification of the observed inductive behavior at 2.9 and 2.7 V. (d) Capacitance vs frequency for various bias voltages exhibits a region of negative capacitance. Reprinted from Bisquert, J.; Garcia-Belmonte, G.; Pitarch, A.; Bolink, H. Negative capacitance caused by electron injection through interfacial states in organic LEDs. *Chem. Phys. Lett.***2006**, *422*, 184–191, with permission from Elsevier. Copyright Elsevier (2006). Right column. Impedance spectra for a CdS/CdTe solar cell. (a) Complex plane plot of the impedance at two different forward biases under dark conditions. (b,c) shows a magnification of the observed inductive behavior at −0.8 and −0.6 V. (d) Absolute value of capacitance vs frequency at forward bias. Reproduced with permission from *Nano Lett.***2006**, *6*, 640–650. Copyright (2006) American Chemical Society.

**Figure 5:**
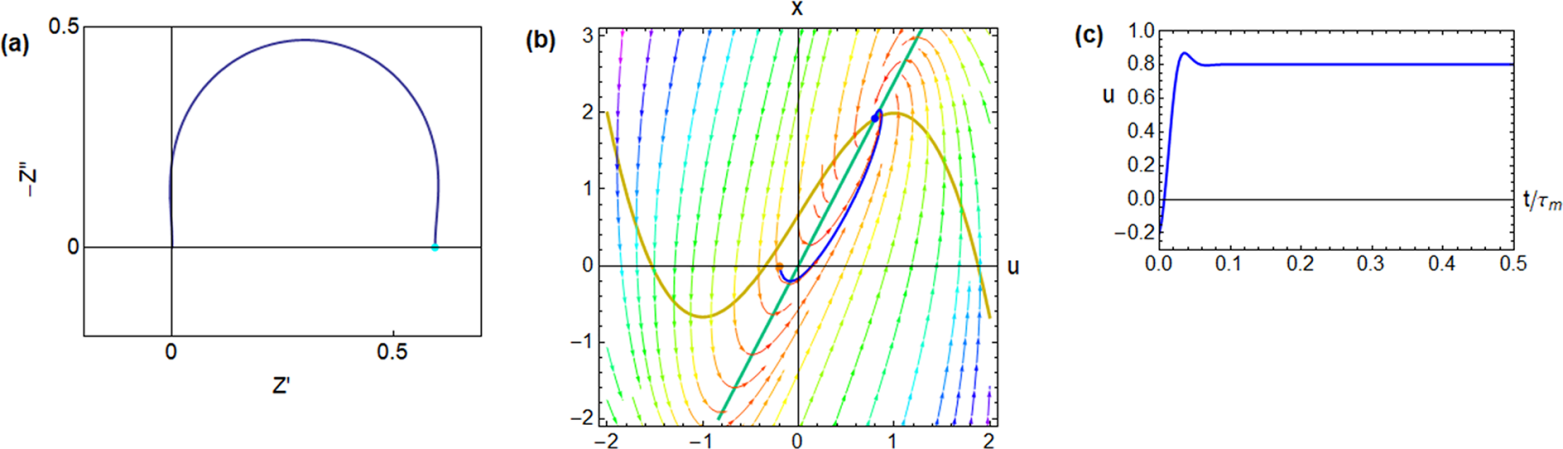
Impedance spectrum and time-domain response of the FitzHugh–Nagumo model dynamical equations,^24^ corresponding to the equivalent circuit of Figure 1b. The model parameters are [*R*_I_, *b*, *r*, ϵ, τ_m_, τ_k_, *u*_app_] = [0.5, 1, 1.2, 2, 0.01, 0.005, 0.8], and the calculated circuit elements and characteristic frequencies are [*R*_a_, *R*_b_, *L*_a_, *C*_m_, ω_a_, ω_b_, ω_L_, ω_c_] = [0.416, −1.39, 0.00208, 0.02, 120, −36.0, 200, 126i] (a) complex plane impedance plot. Green dot: dc resistance. (b) Evolution of a point in the phase plane starting from the orange point, with an external current established at the blue fixed point (voltage *u*_app_). (c) Time evolution of the voltage.

**Figure 6:**
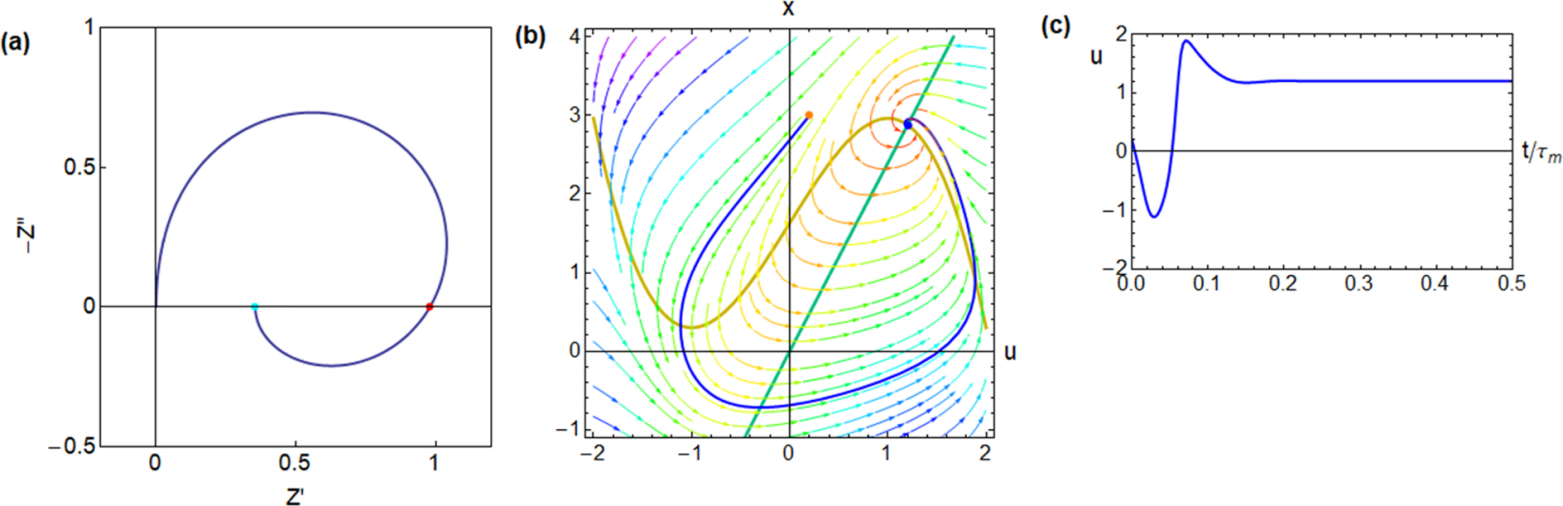
Impedance spectrum and time-domain response of the FitzHugh–Nagumo model dynamical equations,^24^ corresponding to the equivalent circuit of Figure 1b. The model parameters are [*R*_I_, *b*, *r*, ϵ, τ_m_, τ_k_, *u*_Hopf_, *u*_app_] = [0.5, 1, 1.2, 0.3, 0.01, 0.033, 0.837, 1.2], and the calculated circuit elements and characteristic frequencies are [*R*_a_, *R*_b_, *L*_a_, *C*_m_, ω_a_, ω_b_, ω_L_, ω_c_] = [0.417, 1.136, 0.0139, 0.02, 120, 44.0, 30, 52.0] (a) complex plane impedance plot. Green dot: dc resistance. Red dot: resistance at the intercept. (b) Evolution of a point in the phase plane starting from the orange point, with an external current established at the blue fixed point (voltage *u*_app_). (c) Time evolution of the voltage.

**Figure 7:**
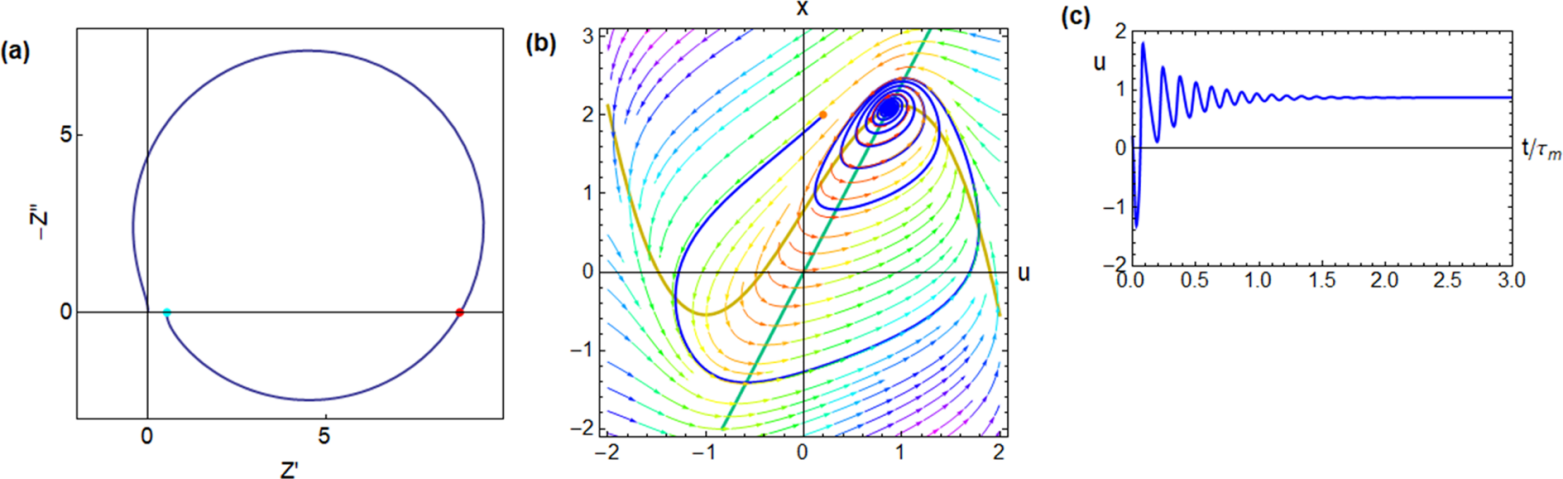
Impedance spectrum and time-domain response of the FitzHugh–Nagumo model dynamical equations,^24^ corresponding to the equivalent circuit of Figure 1b. The model parameters are [*R*_I_, *b*, *r*, ϵ, τ_m_, τ_k_, *u*_Hopf_, *u*_app_] = [0.5, 1, 1.2, 0.3, 0.01, 0.033, 0.837, 0.87], and the calculated circuit elements and characteristic frequencies are [*R*_a_, *R*_b_, *L*_a_, *C*_m_, ω_a_, ω_b_, ω_L_, ω_c_] = [0.417, 1.136, 0.0139, 0.02, 120, 44.0, 30, 52.0] (a) complex plane impedance plot. Green dot: dc resistance. Red dot: resistance at the intercept. (b) Evolution of a point in the phase plane starting from the orange point, with an external current established at the blue fixed point (voltage *u*_app_). (c) Time evolution of the voltage.

**Figure 8:**
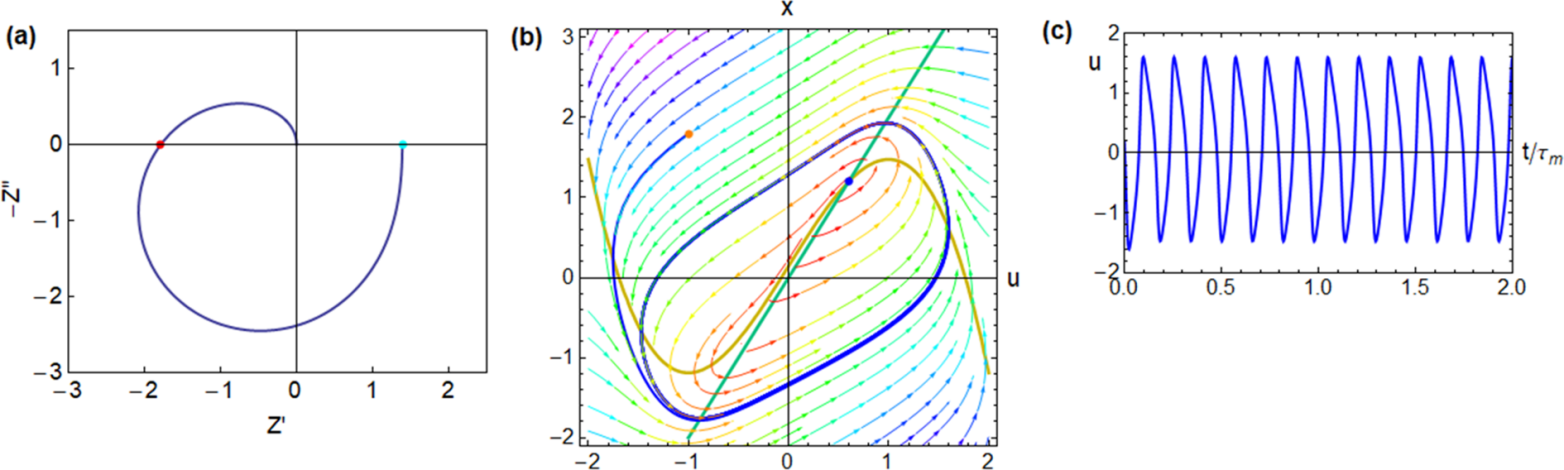
Impedance spectrum and time-domain response of the FitzHugh–Nagumo model dynamical equations,^24^ corresponding to the equivalent circuit of Figure 1b. The model parameters are [*R*_I_, *b*, *r*, ϵ, τ_m_, τ_k_, *u*_Hopf_, *u*_app_] = [0.5, 1.2, 1.2, 0.3, 0.01, 0.033, 0.8, 0.6], and the calculated circuit elements and characteristic frequencies are [*R*_a_, *R*_b_, *L*_a_, *C*_m_, ω_a_, ω_b_, ω_L_, ω_c_] = [0.5, −0.781, 0.0139, 0.02, 100, −64, 36, 48] (a) complex plane impedance plot. Green dot: dc resistance. Red dot: resistance at the intercept. (b) Evolution of a point in the phase plane starting from the orange point, with an external current established at the blue fixed point (voltage *u*_app_). (c) Time evolution of the voltage.

**Figure 9:**
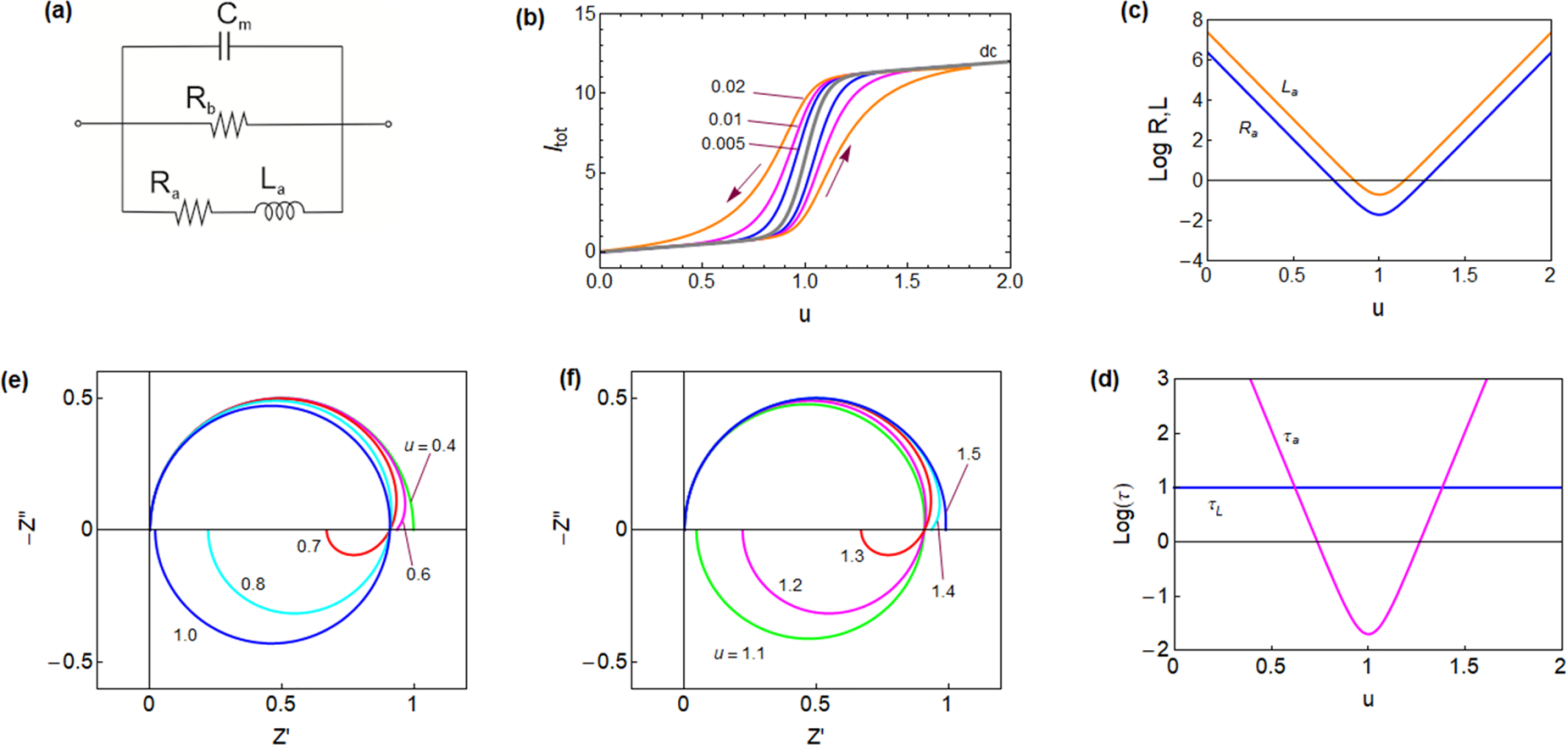
Representation of a model memristor. (a) Equivalent circuit corresponding to eq 8. (b) Current at forward and backward voltage sweeps at the indicated scan rates. The gray line is the stationary (dc) curve. (c) Resistor and inductor as a function of voltage. (d) Time constants τ_a_ = *R*_a_*C*_m_ and τ_L_ = *L*_a_/*R*_a_ as a function of voltage for *C*_m_ = 1; *R*_b_ = 1; *i*_c0_ = 10; *V*_T_ = 1; *V*_m_ = 0.05; and τ_d_ = 10. The crossing points are *u* = 0.62, 1.38. (e,f) Set of impedance spectra at the indicated voltages.

In the first part of the paper, we present the general theory based on a two-dimensional set of differential equations that generate the family of models shown in the equivalent circuits of Figure 1b,d,e. In practical devices and electrochemical systems, the modeling and interpretation are often more complex than these elementary models, as the system contains a variety of features that become manifest in a larger set of differential equations or additional features in the equivalent circuit. However, the presence of the chemical inductor can then be recognized according to the basic structures of Figure 1, which provides important insights into widely observed phenomena such as negative capacitance or self-sustained oscillations. We will also connect the basic dynamical structure with the actual interpretation of experiments. In Section 2.2, we describe the properties of a particular model for a halide perovskite memristor so that we can track the changes of the circuit elements when the voltage is modified, interpret the corresponding spectra, and analyze the connection to time-domain measurements such as the cyclic voltammetry. In Section 2.3, we describe in detail a variety of systems that show the phenomenon of the chemical inductor, and in Section 2.4, we show the self-oscillating systems.

## 2 Results and Discussion

### 2.1 Model and Dynamical Properties

#### 2.1.1 Time-Domain Model and Impedance Analysis

Consider the voltage *u* and current *I* across the device, and an additional internal current denoted by the variable *x*. The system is described by the nonlinear coupled dynamical equations
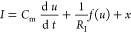
3
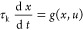
4

The first equation shows that the current *I* is composed of three branches: a capacitive charge with capacitance *C*_m_; a conduction channel of conductivity function *f*(*u*); a resistance scale parameter *R*_I_; and a slow recovery current that responds to the changes by a voltage-driven adaptation function *g*(*x*, *u*), as indicated in the second equation.

The first two channels in eq 3 are “fast” in the sense that the charging time constant τ_u_ = *R*_I_*C*_m_ is much shorter than the adaptation current time constant τ_k_. The steady-state current–voltage characteristic has the form
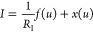
5where the last summand is the solution of *g*(*x*, *u*) = 0. By a linear expansion of eqs 3 and 4 where the small perturbation is denoted by *ŷ,* we obtain the results
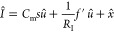
6
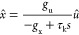
7

Hence, the impedance takes the form
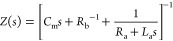
8

This function corresponds to the equivalent circuit of Figure 1b. The circuit elements are defined as^12^
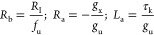
9

The three separate branches of linear impedance are associated with the terms of eq 6 already discussed: (1) the capacitive and (2) conductive channels, and (3) the *RL* branch generated by the delay in eq 4 that becomes 7 in the linearized form. Structurally, the inductor element cannot stand alone in the equivalent circuit due to its origin in a relaxation equation for the slow variable. This result shows that eqs 3 and 4 generate a chemical inductor, whatever the form of the adaptation function *g*(*x*, *u*) or its physico-chemical interpretation. We note that the signs of the partial derivatives in eq 9 have the effect of forming positive or negative circuit elements, which has an important influence on the stability properties as described later.^13^

The model of eqs 3 and 4 is the “backbone” of the emergence of a chemical inductor. More complex systems containing this type of structural dynamics will show one or more chemical inductors. One may use a more general coupling *F*(*u*, *x*) in eq 3, and the linearized equation will have the same form as 6 with different coefficients.

The analysis of the impedance spectra is facilitated^13^ by introducing the characteristic frequencies ω_a_ = (*R*_a_*C*_m_)^−1^, ω_b_ = (*R*_b_*C*_m_)^−1^, and ω_L_ = *R*_a_/*L*_a_ and the correspondent characteristic times τ_a_ = ω_a_^–1^, τ_b_ = ω_b_^–1^, and τ_L_ = ω_L_^–1^. Then, the impedance function 8 can be written as
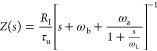
10

A set of impedance spectra for the circuit of Figure 2a obtained by eq 8 is shown in Figure 2b, where *Z*′ = Re(*Z*) and *Z*″ = Im(*Z*). In this figure, only the inductor *L*_a_ is varied. When it is small, the channel *R*_a_*L*_a_ of the equivalent circuit is highly conducting at all frequencies, and the spectrum is simply a positive arc. However, when *L*_a_ increases, the channel is conducting only at very low frequencies. Then, the impedance at a high frequency is the arc *R*_b_*C*_m_ and enters the fourth quadrant, forming the inductive loop and reaching the dc resistance at a low frequency
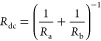
11

Already in 1941, the inductive pattern of Figure 2b was reported for impedance spectroscopy measurement of the squid giant axon by Cole and Baker,^14^ as shown in Figure 3.

A calculation of the frequency of intercept of the real axis (in addition to the obvious points ω = 0, ∞) gives the following result^8^
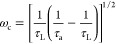
12

Therefore, a crossing of the real axis, the red point in Figure 2b, is observed only if ω_c_ is real, when τ_L_ > τ_a_. The intersection of the characteristic times in Figure 2c corresponds to the value that produces a crossover to the fourth quadrant, see also Figure 9. In some research areas, it is customary to represent the capacitance that corresponds to the impedance data set, see Figure 4. The complex capacitance *C*(ω) is defined from the impedance as *C*(ω) = 1/[iω*Z*(ω)], and the real part is denoted as *C*′(ω) = Re[*C*(ω)]. By eq 2, the effective capacitance of the inductor is
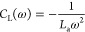
13

It follows from eq 13 that the inductive arc in Figure 2b causes a negative value of Re(*C*) at a low frequency, as shown in Figures 2d and 4d (left); that is a normally denominated “negative capacitance effect”.^4,5,9,15−21^ This effect has been broadly studied in emerging solar cells, as discussed in Section 2.3.4.^4,9,19,20,22^ We remark that the inductive feature commonly found in solar cell devices does not require negative parameters in the equivalent circuit: it is generated by a positive chemical inductor. In the analysis of LEDs and solar cells, it is often represented that the value is Abs[*C*′(ω)], see Figure 4d (right). Then, the crossover to the negative capacitance appears as a spike at the angular frequency ω_c_.

**Figure 10:**
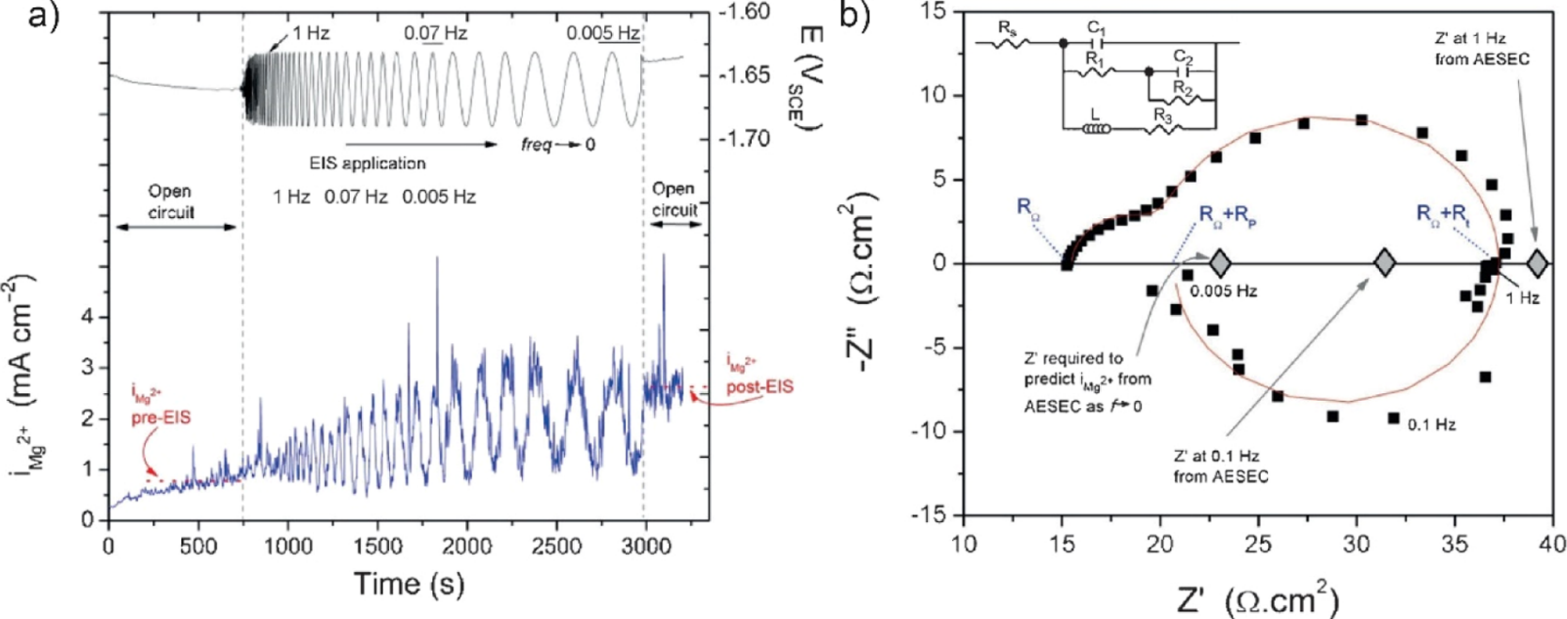
Impedance plot of pure Mg in 1.0 *m* NaCl. Symbols represent experimental EIS data, and the line (in red) represents the fit to the equivalent circuit given in the inset. Reproduced with permission from *ChemPhysChem***2015**, *16*, 536–539. Copyright (2015) John Wiley and Sons.

**Figure 11:**
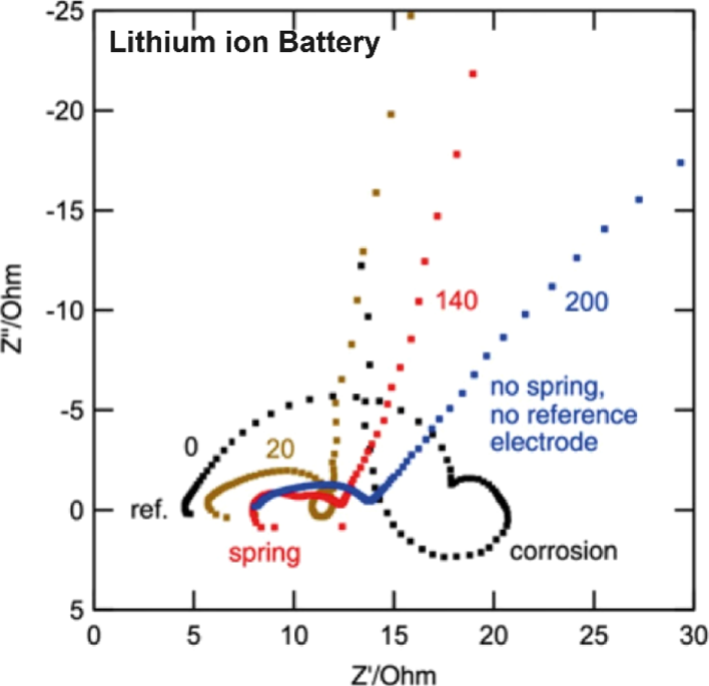
Impedance plot of discharged lithium-ion half cells with graphite. Numbers indicate the charge–discharge cycles carried out. Inductive loops can be switched on and off by adding and removing the spring and the reference electrode, respectively. Reproduced with permission from Brandstätter, H.; Hanzu, I.; Wilkening, M. Myth and Reality about the Origin of Inductive Loops in Impedance Spectra of Lithium-Ion Electrodes—A Critical Experimental Approach. *Electrochim. Acta***2016**, *207*, 218–223. Copyright (2016) Elsevier.

**Figure 12:**
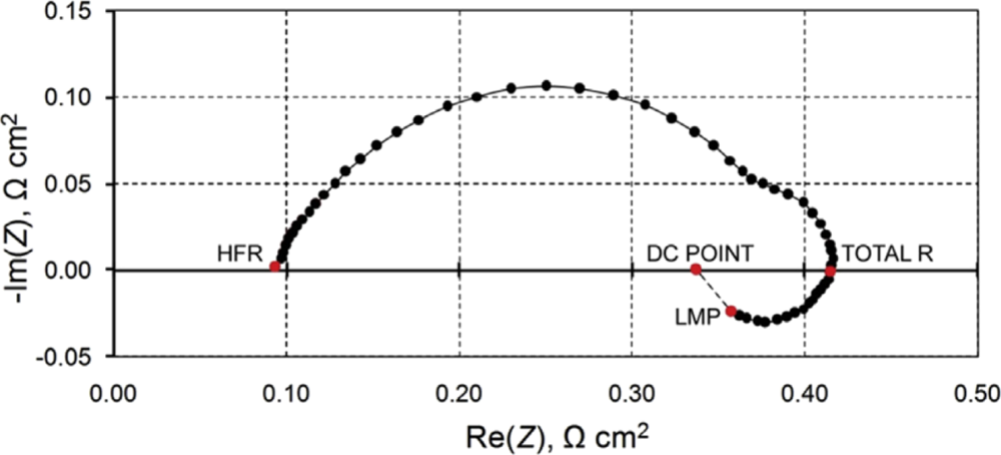
Impedance plot of a H_2_/air PEM fuel cell (50 cm^2^, 65 °C, 0.5 bar(g), 15 A, H_2_/air stoichiometry 2/4, 100% RH), high-frequency resistance, total *R*, DC point, and the last measured point. Reprinted from Pivac, I.; Šimić, B.; Barbir, F. Experimental diagnostics and modeling of inductive phenomena at low frequencies in impedance spectra of proton exchange membrane fuel cells. *J. Power Sources***2017**, *365*, 240–248, with permission from Elsevier. Copyright (2017) Elsevier.

**Figure 13:**
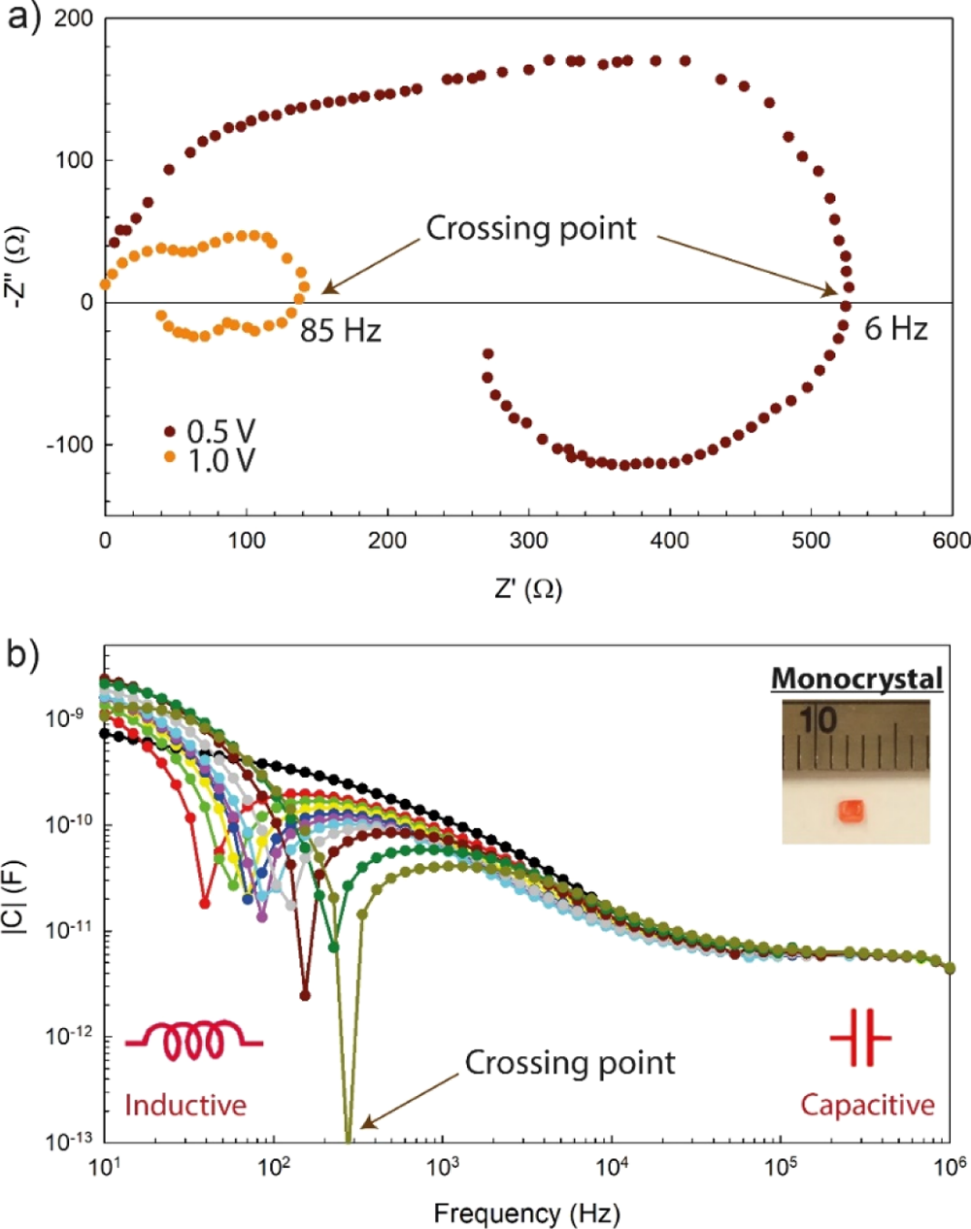
(a) Impedance plot of CH_3_NH_3_PbBr_3_ perovskite single crystals exhibiting the low-frequency inductive (negative capacitance) response. It is observed that the inductive behavior is more featured for higher potentials. (b) Variation of the capacitance absolute value with the bias voltage. The spike corresponds to the crossing of negative values at low frequencies. The 0 V spectrum without induction is marked in black. At high frequencies, responses collapse to the geometrical capacitance. Reprinted from Kovalenko, A.; Pospisil, J.; Krajcovic, J.; Weiter, M.; Guerrero, A.; Garcia-Belmonte, G. Interface inductive currents and carrier injection in hybrid perovskite single crystals. *Appl. Phys. Lett.***2017**, *111*, 163504, with the permission of AIP Publishing. Copyright (2017) AIP Publishing.

#### 2.1.2 Time-Domain Response, Stability, and Self-Sustained Oscillations

There is a direct connection between the impedance properties and the time-domain response. This is amply exploited in the methods of engineering control.^23^ However, electroactive and photoactive materials show a large change in characteristic frequencies as the voltage is varied, which makes the analysis challenging. In the following, we analyze the dynamical effects of the chemical inductor by solving eqs 3 and 4 for a particular model, the FitzHugh–Nagumo neuron (FHN) model, according to a recent report.^24^ A program for the calculation of these features is available online.^25^ This dynamical system is a broadly studied two-dimensional neuron model^6,26−31^ that becomes very rich by the presence of a negative capacitance in *R*_b_ of Figure 1b in addition to the capacitor and inductor. It shows a sudden transition to an unstable regime in which the non-linear system performs self-sustained oscillations, termed a Hopf bifurcation.^32,33^ These are complex properties that will be very briefly described here to show the consequences of the inductor in the dynamics. A full description of impedance spectra and the correspondent dynamics in a variety of systems according to the characteristic frequencies and bifurcations is presented in a separate publication.^13^

The FHN model^26,27^ imitates the generation of action potentials of the more complex HH model with a single recovery variable. It is described by the functions *f*(*u*) = *u*^3^/3 – *u* and *g*(*u*, *x*) = *u*/*R*_w_ – *bw*, where *R*_w_ and *b* are constants. Even with a two-dimensional structure, the bifurcation and dynamical properties are rather complex.^6,29−31^ Recently, the ac impedance properties of the FHN model have been characterized,^24^ and the main results are shown in Figures 5–8.

Here, we discuss some cases of the FHN model when the system is fixed to a certain current associated with the voltage *u*_app_. For each case, we show the associated impedance spectrum, which is an instance of Figure 1b. The phase portrait shows the evolution in the plane of the two variables (*u*, *x*) for the indicated initial condition. The vector field (*u̇*, *ẋ*) gives the possible trajectories in the phase space (*u*, *x*). The nullclines are the lines *u̇* = 0 and *ẋ* = 0, and their intersection is a fixed point. Finally, the projection into the *u*-axis shows the temporal evolution of the voltage.

At a large voltage, the fixed point is stable and the trajectory in the phase plane spirals to the fixed point so that the voltage affects an underdamped oscillation to the steady-state value. This is the case in Figures 5–7. In Figure 5, the inductance is small, and the system is dominated by the *RC* arc in the first quadrant of Figure 5a. The temporal response in Figure 5c is a positive overshoot. In Figure 6 the inductor *L*_a_ is larger than in Figure 5, and the inductive loop becomes fully developed in the fourth quadrant of Figure 6a producing a negative capacitance effect. The time-domain response in Figure 6c shows a negative spike that is due to the nonequilibrium initial situation represented by the initial value of the slow variable *x* = 3, as shown in Figure 6b. In Figure 6c, we note that the combination of the capacitor and inductor produces overdamped oscillations before the system settles to the final voltage *u*_app_. The damped oscillations become much larger in Figure 7, when the system approaches the voltage of the Hopf bifurcation, *u*_Hopf_.

To determine the condition of bifurcation, we develop the linear stability analysis of eqs 3 and 4 based on the Jacobian of 6 and 7.^32^ As the impedance is obtained from the same linearized equations, the elements of the Jacobian can be written in terms of equivalent circuit elements in the form
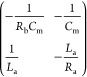
14

The Hopf bifurcation of this nonlinear system happens when the trace of the Jacobian matrix is zero, corresponding to the condition
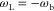
15that is satisfied if *R*_b_ takes negative values. At the bifurcation, the impedance spectrum jumps to the left side of the vertical axis as shown in Figure 8a, presenting a negative resistance value at the frequency ω_c_ (red point). This type of spectrum indicates the occurrence of self-sustained oscillations in neurons and electrochemical systems at a fixed current (galvanostatic conditions), and it was termed as the “hidden negative resistance” by Koper.^1,24,34^ The voltage oscillations are shown in Figure 8c corresponding to the periodic stable trajectory in the phase plane. In Figure 8b is depicted a limit cycle in which, in contrast to the previous figures, each oscillation never passes through the fixed equilibrium point but remains far from equilibrium on the way toward equilibrium.

#### 2.1.3 Potentiostatic Oscillations

If the system is fixed potentiostatically, the voltage between the outer contacts, *V*, is constant, and the circuit in Figure 1b cannot show oscillations. The effect of the series resistance *R*_s_ that is present in electrochemical cells and in all practical solid devices changes the situation as the outer voltage and can be expressed as
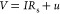
16where *u* is the internal voltage or the potential drop across the double layer. Now, *u* can oscillate since the variations are compensated by the complementary voltage across *R*_s_.^1,35−37^ Combining 16 with 3 and 4, another equivalent circuit structure in the series mode appears as shown in Figure 1d. Note that here, *R*_b_ of Figure 1b is not a necessary element since negative *R*_a_ can be compensated by the positive *R*_s_ to provide the oscillating pattern of Figure 8c. The series model is applied not only in electrochemistry but also in semiconductor device models.^38,39^

Another aspect worth mentioning, as explained by Fletcher,^40^ is that in a typical three-terminal electrochemical cell (with the working, counter, and reference electrodes), inductive and capacitive artifacts appear when the circuit is reduced to a two-terminal impedance, even though the system contains no inductor at all.

In summary, eqs 3 and 4 represent the basic structure of electrical and electrochemical dynamical systems, leading to a chemical inductor. These types of models form a part of the general framework of fast–slow dynamical models,^41^ which includes notorious dynamical models like the van der Pol oscillator or the FitzHugh–Nagumo neuron model.^26,27^ In electrical devices such as solar cells, *u* is the voltage difference between the contacts. In electrochemical systems, *u* is the voltage across the double layer. In biological systems such as neurons,^7^*u* is a transmembrane electrochemical potential that governs ion fluxes. On the other hand, variable *x* is strongly system-dependent and various specific cases are found. It may correspond not only to a slow current effect as already said but also to the activation of ionic conductivity of ion channels in the neuronal membrane,^3^ to surface adsorption,^34,42^ or to the distribution of particles inside a semiconductor device.^39^

#### 2.1.4 Current-Controlled Recovery

We consider a similar dynamical model in which the change of the slow variable is driven by the electrical current. The dynamical equations take the form
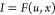
17
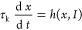
18

The first reported memristor^43^ was described by a model of this type. A linear response analysis provides the impedance in the form
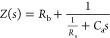
19

This function corresponds to the equivalent circuit of Figure 1e. The circuit elements are
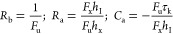
20

For simplicity, we have dropped in eq 17, the main capacitive charging *C*_m_, which may be added as a parallel capacitor for the interpretation of experiments.

To illustrate the model, we consider a simple coupling *F*(*u*, *x*) = *f*(*u*)/*R*_I_ + *x* and *h*(*x*, *I*) = *aI* – *bx*, where *a* and *b* are positive constants. The circuit elements have the values
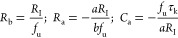
21

This result indicates that in the current–control recovery, there is no chemical inductor but both *R*_a_ and *C*_a_ are negative elements. The remarkable feature is that, despite the negative values that cause impedance components in the third quadrant of the complex plane, the time constant for recovery *R*_a_*C*_a_ = τ_k_/*b* is positive. This is because the adaptation current *x* tends to the value *aI*/*b* as time tends to infinity.

### 2.2 Application of the Impedance Model and Hysteresis: The Case of the Memristor

A memristor is a two-terminal device that undergoes a voltage-controlled conductance change.^44^ There are a variety of material platforms for memristive devices including silicon oxides,^45^ silicon nitrides,^46^ metal oxides,^47,48^ and halide perovskites.^49−51^ These devices are attractive for memory applications and for formation of artificial synapses for brain-inspired computing systems.^52−54^

The resistive switching property and strong hysteresis effect occur because the resistance depends on the history of one or more of the state variables of the system. Therefore, eqs 3 and 4 represent the basic dynamical equations of a voltage-controlled memristor, while a current-controlled model is described by 17 and 18.^44,55^ We can conclude that any memristor of types 3 and 4 will show a chemical inductor effect, as has been shown recently.^12^ Originally, Chua proposed^56^ that the memristor constitutes a fourth fundamental element, but this assumption has been criticized,^57^ since the memristor is a nonlinear element, similar to a diode or a transistor, but in terms of linear response, it can be constructed from the standard elements as the equivalent circuit of Figure 1b.

#### 2.2.1 Impedance Response of a Model Memristor

To illustrate the practical applicability of the concept of the chemical inductor, we show a specific model for a halide perovskite memristor that has been described recently.^58^ It is formed by the dynamical equations
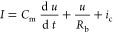
22
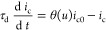
23

The fast variable is the voltage *u,* and the slow variable is the current *i*_c_. The *i*_c_ in equilibrium (d *i*_c_/d *t* = 0) rises from zero to a saturation value *i*_c0_ according to the occupation function θ(*u*) = [1 + e^–(*u*–*V*_T_)/*V*_m_^]^−1^ that satisfies 0 ≤ θ ≤ 1, where *V*_T_ is an onset voltage and *V*_m_ is an ideality factor with a dimension of voltage, see the central gray line in Figure 9b. Eq 23, where τ_d_ is a characteristic time for diffusion, expresses the delay of the current *i*_c_ in reaching the occupation value imposed by the external voltage due to ionic motion that is necessary to form the high conduction state. The equivalent circuit has the standard form of the chemical inductor arrangement, Figure 9a, with a constant capacitance *C*_m_, an Ohmic resistance *R*_b_, and a voltage-dependent resistor and an inductor given by the expressions
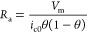
24
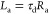
25

Both functions display a minimum at *u* = *V*_T,_ as shown in Figure 9c.

The time τ_L_ = τ_d_ is a constant, and τ_a_ = *R*_a_*C*_m_ is a function of voltage, as shown in Figure 9d. Figure 9e shows the evolution of the impedance spectra as the voltage increases. The high-frequency arc in the first quadrant is barely affected by the voltage changes, but the low-frequency arc in the fourth quadrant undergoes important changes. This is because both *R*_a_ and *L*_a_ become smaller, and the pathway through the *RL* line in the equivalent circuit is activated. The crossing of the characteristic times in Figure 9d indicates that the spectrum enters the fourth quadrant at *u* = 0.62, and the dc resistance becomes minimum at *u* = *V*_T_. When the voltage increases, *R*_a_ and *L*_a_ start to increase and the inductive features recede, as shown in Figure 9f. At *u* = 1.32, the inductive arc vanishes and leaves only the *RC* arc in the first quadrant. Experimental results of the impedance spectroscopy analysis of the halide perovskite memristor show the large chemical inductor effect near the threshold voltage for the transition to the conductive state as shown in refs (21) and (58).

#### 2.2.2 Inductive Hysteresis

An important method to determine the time-domain response of a device or an electrochemical cell is to sweep the voltage at a constant rate, *v*_r,_ as defined by the voltage dependence on time
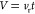
26

This is the method to measure current–voltage curves in solar cells, and it is called cyclic voltammetry in electrochemistry. An example is shown in Figure 9b by calculating the dynamical behavior of 22 and 23 under constraint 26.^58^ The current measured at infinitely slow steps is the gray line, but fast sweeps lead to substantial differences in the forward and reverse scan currents. These hysteresis effects are very significant for the temporal behavior of electronic devices such as solar cells.^59−64^ We have previously described the connection of the hysteresis properties to the equivalent circuit of impedance spectroscopy.^59^ The capacitive current in a forward scan (*v*_r_ > 0) gives an added positive current to the steady-state value^65^
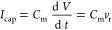
27

The addition of the capacitive current to the equilibrium current makes the forward current larger than in reverse, and this behavior is known as normal hysteresis.^66,67^ On the other hand, the opposite behavior was observed^68^ in which the forward scan decreases the dark current, as shown in Figure 4b, and it is called inverted hysteresis.^61−63^ Recently, it was shown that inverted hysteresis is produced by the low-frequency inductor.^59,64^ This is because the effective total capacitance is negative, as shown in eq 13; hence, the sign of the transient current changes. In terms of eqs 22 and 23, the transient inductive current has the form
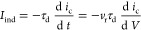
28

It is proportional to the scan rate, just like the capacitive current, but it is negative in the forward scan since d *i*_c_/d *V* > 0. This is the general reason why normal hysteresis is capacitive and inverted hysteresis is inductive.^59^

### 2.3 Non-Oscillatory Systems that Exhibit a Chemical Inductor

Let us review systems in which the chemical inductor causes important effects, starting with the systems that do not oscillate by the absence of bifurcations. In general, these systems fulfill all the requirements of eqs 3 and 4. For example, electrochemical reactions that respond to an externally applied voltage–current (fast process) require the supply/departure of ions coupled from the catalytic sites (slow process). A wide range of systems fulfill the requirements such as in the electrochemical corrosion of metal alloys, proton membrane fuel cells, solar cells, memristors, and LEDs.

#### 2.3.1 Corrosion of Metal Alloys

In the field of metallurgy, corrosion of alloys has been widely studied by impedance spectroscopy and observation of the impedance loop has been reported in the presence of reactive metals.^69^ Recently, IS measurements have been coupled with atomic emission spectroelectrochemistry to monitor onsite the electrochemical dissolution of Mg^2+^.^70^ It is observed that the oxidation of the metal Mg(0) to form Mg^2+^ correlates with the applied voltage perturbation introduced during the IS measurement (Figure 10a), and the IS spectrum shows the presence of the inductive loop (Figure 10b). The combination of the two techniques allows us to conclude that dissolution of Mg^2+^ ions is the slow and limiting step in this electrochemical reaction and must be the slow process of eqs 3 and 4. Indeed, during the frequency sweep at frequencies below 1 Hz, the voltage excitation becomes slow enough to enable all Mg^2+^ ions to depart from reactive centers, leading to the formation of the inductor. Of course, the kinetics of dissolution could be modified if conditions are such that they are no longer mass-transport limited. For example, by using efficient stirring or by sonication of the reactive surface. Overall, the presented model in this work could be applied to obtain accurate kinetic information about the corrosion process. It is noted that the high-frequency response is more complex than that arising from eqs 3 and 4 as it shows two arcs, and further additions need to be introduced into the model.

#### 2.3.2 Corrosion of Electrodes in Batteries

Metal alloys are used as structural elements in electrodes for several electrochemical systems with solid/liquid or solid/solid interfaces like in batteries. One can infer that if the alloy contains a reactive metal under the operating conditions, corrosion will also be observed in the complete device and the inductive loop will appear. This is clearly the case for the emerging Mg–air batteries where Mg–Al–Pb alloys are used as anode electrodes.^71^ It was shown in the previous section that the slow Mg^2+^ dissolution acts as the slow process required to observe the inductive loop. Not surprisingly, batteries containing this alloy also show the inductive loop at low frequencies as a clear sign of corrosion.

The actual origin of the observed inductances for Li-ion batteries has been recently reviewed.^72^ For a long time, the formation of the solid electrolyte interphase was held responsible for the presence of the inductive loop. Although not totally ruled out, the authors identified four other different sources of chemical inductance loop formation that include external parameters such as the actual setup for the experiment (Figure 11). For example, the use of springs in the Swagelok T-cell does generate a magnetic inductance at high frequencies; the cell not being measured in the steady-state (drift) leads to formation of a loop at an intermediate frequency, and corrosion of the electrodes and/or the reference electrode is observed in the low-frequency region.

#### 2.3.3 Proton Membrane Fuel Cells

Proton-exchange membrane (PEM) fuel cells often lead to inductive loops (Figure 12).^73−75^ The reasons for the observation of the inductance have been reviewed recently, but the interpretation of the impedance spectra at low frequencies is still ambiguous. Several mechanisms have been proposed such as side reactions with intermediate species, carbon monoxide poisoning, and/or issues with water transport. On the one hand, inductive loops have been predicted by models that account for formation of hydrogen peroxide as an intermediate in a two-step oxygen reduction reaction. Similarly, this oxidant can be a source of corrosion for the Pt contact that leads to dissolution of the metal.^74^ Overall, in this electrochemical system, there is more work to be done to understand the chemical origin of the inductive loop, but our model could help to identify the nature of these processes.

#### 2.3.4 Solar Cells and Light-Emitting Diodes

Emerging solar cells, made by combinations of organic and inorganic materials and liquid electrolytes, have been broadly studied by impedance spectroscopy,^8,76,77^ and the presence of the chemical inductor is widespread. In 2006, a broad variety of solar cells containing a “negative capacitance” were analyzed^4^ such as nanowired ZnO/CdSe/CuSCN, thin-film CdS/CdTe, and dye-sensitized solar cells, and the equivalent circuit of Figure 1b was found to provide a good description of the impedance response, Figure 4a (right). The same inductive impedance pattern is found in organic LEDs as they share device configuration and materials, as shown in Figure 4a (left).^5^

Lead halide perovskite solar cells are a recent class of solution-processed hybrid photovoltaic devices with outstanding efficiencies.^78^ These solar cells have shown intense inductive features in very early reports,^79,80^ and the features have been confirmed in measurements of stabilized and robust devices.^9,19,20,64,81^ An example of a perovskite single crystal is shown in Figure 13. At 0 V of applied bias (black data points), there is no inductive feature, as the curve in Figure 13b has no spike. However, at 0.5 V and 1 V, the chemical inductance is clearly appreciated as a double inductive feature in Figure 13a, which has also been observed in halide perovskite memristors.^21^ The inductive effect has been associated with the presence of mobile ions that cause a number of effects in the halide perovskites.^8,81^ The slow mode that generates the inductor line in the equivalent circuit has been interpreted as a delayed surface voltage^82^ and as a sluggish surface recombination current.^22,83,84^ It has been shown that the inductive impedance is correlated to the amount of inverted hysteresis in current–voltage curves,^59,64^ as described in Section 2.2.2.

### 2.4 Oscillating Systems

#### 2.4.1 Electrochemical Oscillators

**Figure 14:**
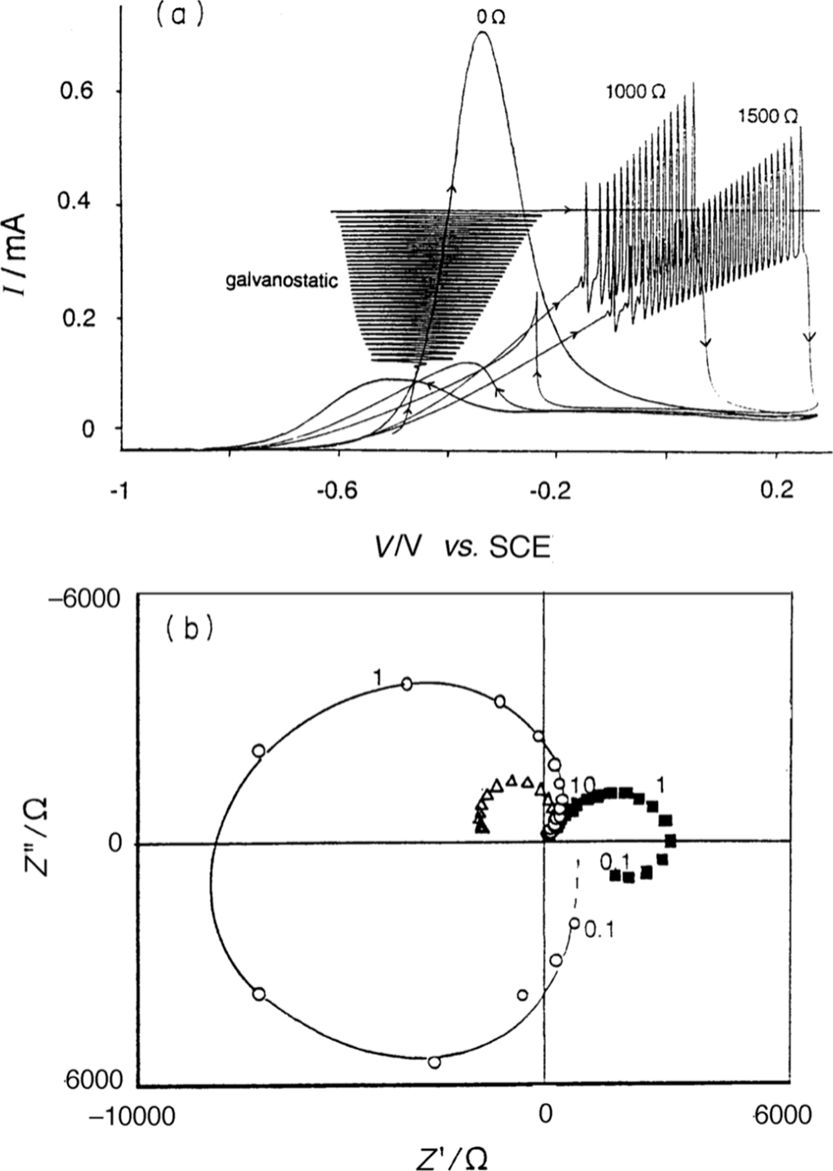
(a) Voltammogram of 0.1 M HCHO in 0.1 M NaOH for a 0, 1000, and 1500 Ω external resistance (internal cell resistance ca. 95 Ω). Scan rate 10 mV s^–1^, 3000 rev min^–1^. Amperogram taken at 0.01 mA s^–1^. (b) Impedance diagrams taken at −0.50 V (■), −0.45 V (○), and −0.35 V (Δ). Indicated frequencies in Hz. Republished with the permission of Royal Society of Chemistry, from Koper, M. T. M. Nonlinear phenomena in electrochemical systems. *Journal of the Chemical Society, Faraday Transactions***1998**, *94*, 1369. Copyright (1998) Royal Society of Chemistry.

The mechanism of the inductor is well known in electrochemistry in relation to the electrochemical impedance spectroscopy of reactions with an intermediate adsorbed species or with an autocatalytic step.^42,85−87^ Some electrochemical systems with chemical inductor structures oscillate, and others do not, as shown in Section 2.3, depending on the conditions of Hopf bifurcations that were stated in Section 2.1.2. The self-sustained oscillations in electrochemical systems^1,35−37^ have been fully classified using the theory of bifurcations, stability, and the methods of impedance spectroscopy.^88−92^ The presence of oscillations in a two-dimensional system requires a negative resistance domain. These systems are usually characterized on the basis of equivalent circuits that combine Figure 1b,c, often with more complex features corresponding to the set of couplings in the reaction mechanism, according to the general theory of chemical oscillators.^32^Figure 14b shows the realization of the impedance spectra of Figures 6 and 8, and the associated oscillations are shown in Figure 14a.^92^ A famous representative model is the system developed by Koper and Sluyters^34^ that considers a single species which diffuses toward the electrode where it is successively adsorbed and electrochemically oxidized.^89,93^ A detailed analysis of the impedance properties of this model is presented in our recent work.^13^

#### 2.4.2 Excitability and Spiking of Neurons

**Figure 15:**
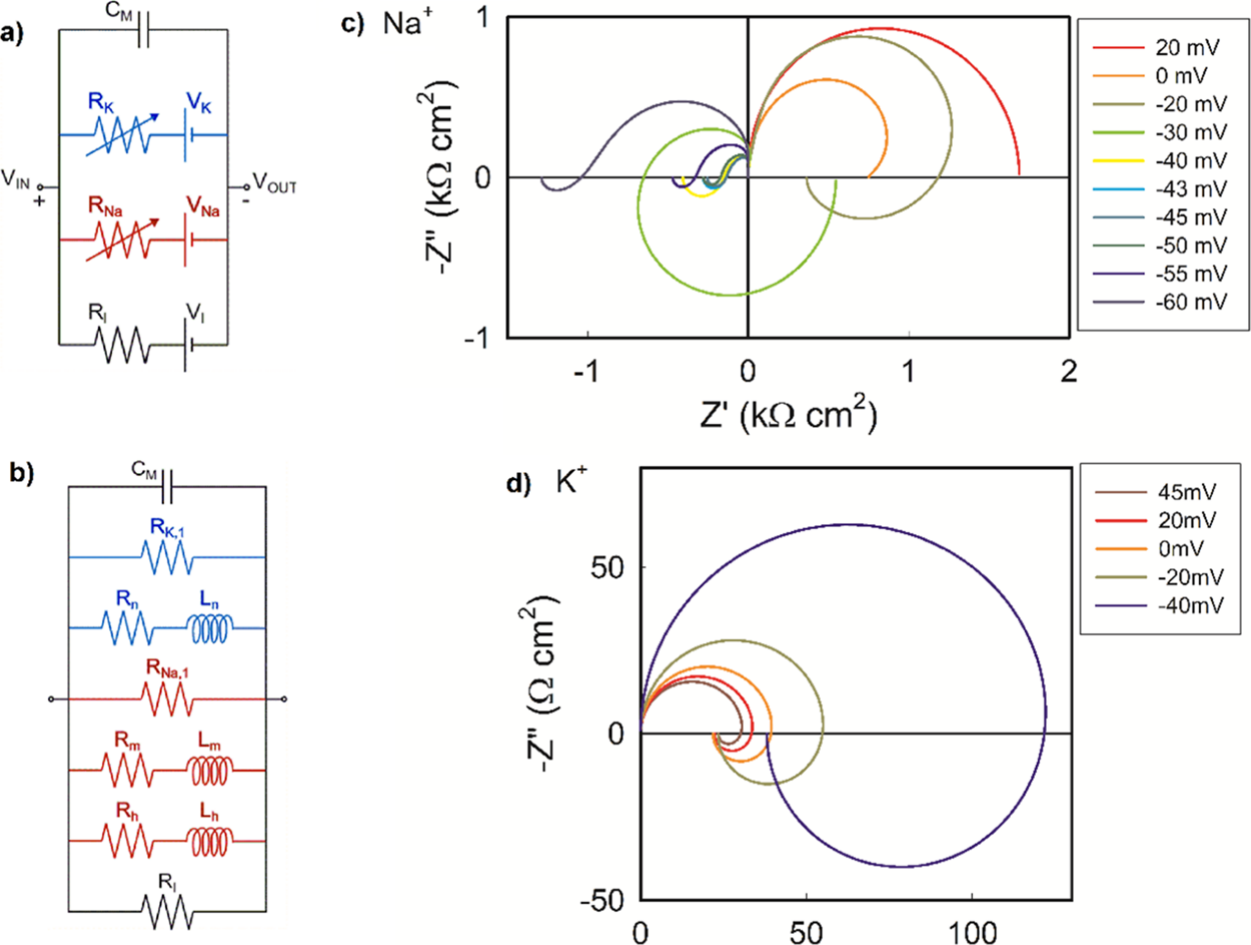
(a) Hodgkin–Huxley electrical model for the squid giant axon membrane consisting of variable resistances in the ion channels as defined in the original publication. (b) Equivalent circuit for the Hodgkin–Huxley model for small ac voltage perturbations. The potassium channel components are indicated in blue and the sodium elements in red. Impedance complex plane plots of the HH model for different membrane voltages for (c) sodium channel and (d) potassium channel. Adapted with permission from Bou, A.; Bisquert, J. Impedance spectroscopy dynamics of biological neural elements: from memristors to neurons and synapses. *J. Phys. Chem. B***2021**, *125*, 9934–9949. Copyright (2021) American Chemical Society.

In neuron ensembles, the phenomenon of excitability is controlled by a Hopf bifurcation in which the neuron makes a transition from a resting state to a rhythmic oscillation characterized by rich spiking patterns that form the basis of computation in the natural brain.^6,7,94^ The stimulation of the neuron transmembrane voltage causes several selective molecular membrane channels to open and close, allowing many ionic and molecular substances to flow and creating an action potential or a spike of 100 mV that is repeated with periodic rhythms.^95^ The central framework for the understanding and physical characterization of neuron excitability is the model of Hodgkin and Huxley (HH)^3^ developed around 1950 for the squid giant axon. This dynamical model is more complex than that of eqs 3 and 4 as it contains a four-dimensional structure associated with the voltage and three recovery variables termed [*n*, *m*, and *h*] that determine the voltage-dependent conductances of sodium and potassium ion channels. In the paper of HH, the neuron-equivalent circuit is presented in terms of time-dependent resistances, as shown in Figure 15a, and the inductor does not appear explicitly. However, whenever the small ac impedance of the HH model is calculated,^2,55,96−100^ a circuit of the classes of Figure 1b–d appears, and this is because HH contains the mechanism of the chemical inductor. Early measurements of the neuron impedance are shown in Figure 3, providing a clear instance of the chemical inductor in an oscillating system.

In fact, a complete analysis of the small ac equivalent circuit of HH,^12^ first presented by Cole (p 299),^100^ shows that the potassium channel presents the structure of Figure 1b, see Figure 15b, while the sodium channel contains two chemical inductor branches so that the equivalent circuit contains a total of three inductive branches corresponding to the mentioned recovery variables. The results in Figure 15 of a calculation of impedance responses as the membrane voltage is varied from the resting state show different impedance patterns: in c and d, the impedance of Figure 6a is shown both for sodium and potassium channels. While the potassium channel does not contain a negative resistance, the hidden negative resistance of Figure 8a occurs in a narrow voltage range in Figure 15c for the sodium channel. Here arise the self-sustained oscillations of the giant axon. Furthermore, the sodium channel shows the phenomenon of the negative inductor.^12^

The inductive behavior of non-magnetic origin in the squid giant axon was well recognized before 1940 by Cole, based on impedance spectroscopy measurement as in Figure 3,^2^ but a reasonable interpretation was not obtained. He later remarked that “the suggestion of an inductive reactance anywhere in the system was shocking to the point of being unbelievable.”^100^ Hodgkin and Huxley^3^ proposed that the potassium conductance is proportional to the power of a variable that obeys a first-order equation in order to match the very different transient curves: the delayed increase in depolarization but a simple exponential decay in repolarization. Thus, as explained later by Hodgkin,^101^ “the inductance is mainly due to the delayed increase in potassium conductance, which can make the membrane current lag behind voltage, provided the internal potential is positive to the potassium equilibrium potential.” This is a clear early formulation of a chemical inductor mechanism.

Since the HH model shows a great deal of complexity, simpler models are used that describe well the properties of neural dynamics with a reduced number of adaptative parameters. The minimal structure that generates action potentials is described by a two-dimensional model as shown in eqs 3 and 4.^6,102^ An example of the properties of the FHN model has been described in detail in Section 2.1.2.

#### 2.4.3 Conditions for Self-Sustained Oscillations

As described in Section 2.1, the impedance spectra provide a mark of the underlying dynamic regimes. It is particularly interesting to establish the fundamental structure of systems that show rhythmic oscillations of an external variable. Understanding the impedance properties of neurons is an important tool for building artificial neurons for neuromorphic computation.^103,104^ The analysis of neuronal and electrochemical systems presented earlier in this work can be combined to show the properties of oscillations in terms of equivalent circuit properties, as derived from linear stability analysis. The destabilizing effect of a negative resistance that leads to instability and oscillations^36,105,106^ and the requirement of an inductor to generate action potentials^100,107,108^ have been previously recognized.

According to our analysis, the general features of a two-dimensional system that shows self-sustained oscillations are

(1) membrane or surface capacitance,
(2) a chemical or an electromagnetic inductor, and
(3) in Figure 1b, either *R*_b_ or *L*_a_ must take negative values to satisfy the condition 15 for a Hopf bifurcation that induces a stable limit cycle. A similar condition occurs for the series circuit in Figure 1d.

Living brains do not use integrate-and-fire (RC) neurons, in contrast to many current neuromorphic computational systems. As discovered by Cole and Hodgkin and Huxley, real neurons contain a chemical inductor mechanism that provides the response properties of the action potential.

## 3 Conclusions

We found a common structure in electrochemical, biological, and semiconductor systems in which a fast conduction mode contains a slow channel with time-dependent dynamics. This structure leads to a generic circuit that contains a capacitance and a resistor, and the slow mode gives an inductance that is termed a chemical inductor. When negative elements such as a negative differential resistance come into play, this dynamical model can be generalized to a self-sustained oscillating system under an externally fixed voltage or current. The transition from rest to periodic spiking occurs at a Hopf bifurcation. The experimental analysis of the impedance spectra enables us to recognize the dynamical properties of such systems.
